# Genetic Contributions to the Development of Complications in Preterm Newborns

**DOI:** 10.1371/journal.pone.0131741

**Published:** 2015-07-14

**Authors:** Chiara Poggi, Betti Giusti, Elena Gozzini, Alice Sereni, Ilaria Romagnuolo, Ada Kura, Elisabetta Pasquini, Rosanna Abbate, Carlo Dani

**Affiliations:** 1 Division of Neonatology, Careggi University Hospital of Florence, Florence, Italy; 2 Department of Experimental and Clinical Medicine, University of Florence - Atherothrombotic Diseases Centre, Careggi University Hospital of Florence, Florence, Italy; 3 Department of Information Engineering University of Florence, Florence, Italy; 4 Metabolic and Muscular Unit, Clinic of Pediatric Neurology, Meyer Children's Hospital, Florence, Italy; 5 Department of Neurosciences, Psychology, Drug Research and Child Health, Careggi University Hospital of Florence, Florence, Italy; The Ohio State Unversity, UNITED STATES

## Abstract

**Aim:**

We aimed to identify specific polymorphisms of genes encoding for vascular endothelial growth factor A (*VEGFA*), endothelial nitric oxide synthase (*eNOS*), renin-angiotensin system (angiotensinogen gene [*AGT*], angiotensinogen type 1 receptor [*AGTR1*], angiotensin-converting enzyme [*ACE*]), and heme oxygenase-1 (*HMOX-1*) in a cohort of preterm infants and correlate their presence with the development of respiratory distress syndrome (RDS) requiring mechanical ventilation (MV), bronchopulmonary dysplasia (BPD), intraventricular hemorrhage (IVH) and retinopathy of prematurity (ROP).

**Study Design:**

We carried out a retrospective study to evaluate the allele frequency and genotype distribution of polymorphisms of *VEGFA*, *eNOS*, *AGT*, *AGTR1*, *ACE*, and *HMOX-1* in a population of preterm neonates (n=342) with a gestational age ≤28 weeks according to the presence or absence of RDS requiring MV, BPD, IVH, or ROP. Moreover, we evaluated through the haplotype reconstruction analysis whether combinations of the selected polymorphisms are related to the occurrence of RDS, BPD, IVH and ROP.

**Results:**

In our population 157 infants developed RDS requiring MV, 71 BPD, 70 IVH, and 43 ROP. We found that TC+CC rs2070744 *eNOS* (41.7 vs. 25.4%, p=0.01) and GT+TT rs1799983 *eNOS* (51.8 vs. 35.2%, p=0.01) polymorphisms are independent risk factors for BPD. Haplotype reconstruction showed that haplotypes in VEGF and *eNOS* are significantly associated with different effects on RDS, BPD, IVH, and ROP in our population.

**Conclusions:**

We found that TC+CC rs2070744 *eNOS* and GT+TT rs1799983 *eNOS* polymorphisms are independent predictors of an increased risk of developing BPD. Haplotypes of *VEGFA* and *eNOS* may be independent protective or risk markers for prematurity complications.

## Introduction

There is increasing evidence that some genepolymorphismsare implicated in the development of severe complications of preterm birth, including respiratory distress syndrome (RDS), bronchopulmonary dysplasia (BPD), intraventricular hemorrhage (IVH), and retinopathy of prematurity (ROP).

Vascular endothelial growth factor A (*VEGFA*) is important for the physiological growth of retinal vessels, and experimental and clinical data suggest that VEGF may play a causative role in the development of ROP [1]. Similarly, *VEGFA* plays a central role in vascular lung repair and its absence results in impaired fetal lung microvascular development [2]. Accordingly, clinical studies suggest a relevant association between *VEGFA* activity and BPD development in preterm infants [3,4].

Nitric oxide (NO) has several vascular actions which may contribute to the growth and protection of vessels in preterm infants [5]. This seems to be confirmed by the reported association between endothelial NO synthase (*eNOS*) gene activity and the risk of developing RDS, BPD, and IVH in this population [6].

The renin-angiotensin system (RAS) is important for regulating systemic blood pressure and volume, and its genetic modifications could affect the outcome of preterm infants. Previous studies have reported the possible association between angiotensin-converting enzyme (*ACE*) and angiotensin type 1 receptor *(AGTR1)* polymorphisms and the risk of developing BPD [7–10] and ROP [11–12], but results are still inconclusive.

Heme oxygenase-1 (HMOX-1) is an enzyme degrading heme to iron ions, carbon monoxide, and biliverdin. Products of HO-1 activity perform important physiological functions in the vascular system, which are ultimately linked to the protection of endothelium through cytoprotective, promitogenic, and anti-inflammatory action [13]. The efficacy of this enzyme has been found to be affected by repeat polymorphisms in the promoter of *HMOX-1* gene [13]. Although ample clinical data indicate its influence on cardiovascular complications in adult patients, the possible correlation between *HMOX-1* gene transcription and the outcome in preterm infants has never been investigated.

On the basis of these considerations, we hypothesized that the occurrence of severe complications in preterm infants—RDS requiring mechanical ventilation (MV), BPD, IVH and ROP—might be affected by polymorphisms in genes coding the *VEGFA*, *eNOS* enzyme, RAS system (Angiotensinogen gene [*AGT*], *AGTR1*,*ACE*), and *HMOX-1* enzyme. To assess this hypothesis we aimed to genotype specific polymorphisms of these genes in a cohort of preterm infants and correlate their presence to the development of RDS requiring MV, BPD, IVH, and ROP.

## Materials and Methods

We performed this study following approval of the local medical ethics committee of Careggi University Hospital. Parental consent was not obtained because patient records/information were rendered anonymous and de-identified prior to analysis.

### Study design

We carried out a retrospective study to evaluate the possible relationship between twelve polymorphisms in genes encoding for *VEGFA*, *eNOS*, RAS system, and *HMOX-1* and the occurrence of RDS requiring MV, BPD, IVH and ROP in a population of preterm newborns. By using haplotype reconstruction analysis we evaluated whether combinations of the selected polymorphisms are related to the occurrence of the aforementioned prematurity complications.

### Patient population

We studied 342 preterm neonates with a gestational age ≤28 weeks, admitted consecutively to the Neonatal Intensive Care Unit of the Careggi University Hospital of Florence, from January 2004 to December 2012. Exclusion criteria were the diagnosis of major congenital malformations, inherited errors of metabolism, and any other congenital syndrome. All subjects investigated were of Caucasian origin in order to guarantee a homogenous ethnic background.

The DNA analysis was performed on dried blood spots collected from infants at 48 hours of life for the local screening program [14].

### Clinical Features and Outcome

Birth weight, gestational age, type of delivery, antenatal steroid treatment, gender, Apgar Score at 5 min, RDS occurrence, need of MV and surfactant, type and duration of respiratory assistance [nasal continuous positive airways pressure (NCPAP), patient-triggered ventilation (PTV),high frequency oscillatory ventilation (HFOV)] were recorded for each newborn infant.

The RDS diagnosis was made as previously reported [15]. Infants were given MV when the pH was <7.20, pO_2_ was <50 mmHg with FiO_2_>0.50, and pCO_2_ was >65 mmHg.

We reported the occurrence of patent ductus arteriosus (PDA), sepsis, IVH, periventricular leukomalacia (PVL),BPD, necrotizing enterocolitis (NEC), ROP, and mortality. Diagnosis of BPD was based on the need for supplemental oxygen to maintain adequate oxygenation at36 weeks of post-conceptional age [16]. Intraventricular hemorrhage, PVL, and NEC were diagnosed following Papile’ [17], de Vries’ [18], and Bell’s criteria [19], respectively. ROP was diagnosed following the International Classification criteria [20].

All clinical data were reported on data sheets designed for this study.

### Studied polymorphisms

The criteria for selection of candidate genes in the present study were their potential involvement in the pathogenesis of the aforementioned prematurity complications. The DNA analysis was performed on dried blood spots collected from infants at 48 hours of life for the local screening program using the QIAamp DNA Micro Kit according to the manufacturer’s instructions (QiagenGmbh, Hilden, Germany).

We studied 12 single nucleotide polymorphisms (SNPs) in the 6 candidate genes (*ACE*, *AGTR1*, *AGT*, *eNOS*, *VEGFA*, *HMOX-1*) according to their demonstrated or putative function based on literature data, localization in the functional regions, as extracted from the dbSNP database (http://www.ncbi.nlm.nih.gov/entrez/query.fcgi?db=snp&cmd=search&term=) (Table 1). SNP information was assessed in dbSNP NCBI and ENSEMBL (http://www.ensembl.org/index.html) databases.

**Table 1:** Description of the 12 studied single nucleotide polimorphisms (SNPs) in *ACE*, *AGTR1*, *AGT*, *eNOS*, VEGF and *HMOX1* genes.

Gene Symbol Chr. Position	Reference Sequence Number	db SNP ID number(Taqman assay number)	Common SNP name and nucleotide substitution	Region	Relative Distance
***ACE*17q23.3**	**NG_011648.1**	**rs4291(C__11942507_10)**	-240A>T	5’ near gene(promotor)	0
		**rs1799752 Custom TaqMan SNP Genotyping Assays**	Alu I/D (287 bp)	Intron 16	11696
***AGTR1*3q24**	**NG_008468.1**	**rs5186(C___3187716_10)**	1166 A>C	3’-UTR	0
***AGT*1q42.2**	**NG_008836.1**	**rs699(C___1985481_20)**	803 A>GMet 235 Thr	Exon 2	0
***eNOS***	**NG_011992.1**	**rs2070744 (C__15903863_10)**	-786 T>C	5’ near gene	0
**7q36**					
		**rs61722009**	*eNOS* 4a4b	Intron 4	4147
		**rs1799983(C___3219460_20)**	894 G>TGlu298Asp	Exon 7	5866
***VEGFA***	**NG_008732.1**	**rs1547651(C___8311590_10)**	A>T	5’ near gene	0
**6p12**					
		**rs833058(C___1647396_20)**	C>T	5’ near gene	1210
		**rs833061(C___1647381_10)**	-460 T>C	5’ near gene	6842
		**rs3025039(C__16198794_10)**	936C>T	3’-UTR	21892
***HMOX1*22q13.1**		**rs3074372**	GT repeat	5’ near gene	0

Genotyping of *ACE* gene Insertion/Deletion (I/D) polymorphisms was performed by TaqMan designed assay, according to Koch et al. [21]. The *eNOS* 4a4b polymorphism was evaluated by PCR method, as previously described [22].

The other polymorphisms [*ACE* rs4291; *AGT*Mrs699; *AGTR1* rs5186; *eNOS*rs2070744 and rs1799983; *VEGFA* rs1547651,rs833058, rs833061 and rs3025039] were analyzed by using TaqMan SNP genotyping assays through the real-time PCR (7900HT Fast Real-Time PCR System, Life Technologies Italia, Monza, Italy).

The (GT)n repeat polymorphism in the promoter of *HMOX-1* was detected according to Vazquez-Armenta G et al. [23].

### Data analysis

Statistical analysis was performed using the SPSS package v18.0. Continuous variables were expressed as mean and standard deviation, and categorical variables were expressed as frequencies and percentages. Tests for conformity with Hardy—Weinberg equilibrium were performed using a standard χ^2^ test. Genotype distributions were compared between groups by χ^2^ analysis. We assessed the association between each polymorphism and clinical adverse outcomes (RDS, BPD, IVH, ROP) by using a dominant model of inheritance, which compares individuals with one or two rare alleles (heterozygotes+homozygotes) to a baseline group of homozygous wild type subjects.

The association of the studied polymorphisms with gestational age and birth weight was also evaluated. Comparisons between median values of gestational age and birth weight among genotypes were performed using the non-parametric Mann-Whitney U test. Logistic regression analysis was used to estimate odds ratios (OR) and 95% confidence intervals (CIs). The independent association of polymorphisms with the different clinical endpoints was evaluated by logistic regression analysis adjusted for gestational age and weight at birth. A value of *p* = 0.05 was chosen as the cut-off level for statistical significance.

### Haplotype analysis

In order to perform haplotype reconstruction analysis (to infer the most probable set of polymorphisms of each studied gene that are inherited together from one parent) in our population, data files were processed in R environment (http://www.r-project.org). Haplotype reconstruction and frequency estimation were independently performed using the PHASE v2.1 software [24], and R package haplo.stats by expectation-maximization strategy (EM algorithm). The haplo.stats package was also used to identify statistically significant associations between haplotypes and clinical endpoints by means of generalized linear models: positive or negative coefficient values indicate the risk or the protection conferred by the haplotypes, respectively, and the absolute values of the coefficient indicate the magnitude of the associations. In order to reduce the type I error, we applied the false discovery rate (FDR) multiple testing correction in generalized linear model analysis [25].

## Results

We found that 157 infants developed RDS requiring MV, 71 BPD, 70 IVH, and 43 ROP in our study population (S1 Appendix). Table 2 shows clinical and demographic data of the enrolled preterm infants. Genotype distributions and allele frequencies of twelve polymorphisms investigated in our population are shown in Table 3.

**Table 2:** Demographic and clinical characteristics of enrolled infants. Mean±SD and rate and (%).

Characteristics	n = 342
**Birth weight (g)**	915 ± 249
**Gestational age (wks)**	27.1 ± 1.8
**Cesarean delivery, n (%)**	227 (67)
**Antenatal steroids, n (%)**	240 (71)
**Male, n (%)**	160 (47)
**Apgar score at 5’** ^**th**^ **min**	7.9 ± 0.86
**RDS**	160 (47)
**Mechanical Ventilation**	157 (46)
**Surfactant**	299 (87)
**PDA**	279 (82)
**Sepsis**	86 (25)
**IVH**	70 (21)
**PVL**	12 (4)
**BPD**	71 (21)
**NEC**	9 (3)
**ROP**	43 (13)
**Death**	23 (15)

**Table 3:** Genotype distribution and allele frequency of the studied polymorphisms.

Gene Symbol	dbSNP	Genotypes	n (%)	F	MAF
***ACE***	**rs1799752**	**II**	58 (16.9)		
		**ID**	166 (48.5)		
		**DD**	118 (34.6)		
		**D**		0.59	0.51°
***ACE* Prom**	**rs4291**	**AA**	119 (34.8)		
		**AT**	171 (50.0)		
		**TT**	52 (15.2)		
		**T**		0.40	0.39°
***AGTR1***	**rs5186**	**AA**	211 (61.7)		
		**AC**	108 (31.9)		
		**CC**	22 (6.4)		
		**C**		0.22	0.29°
***AGT***	**rs699**	**MM**	85 (24.9)		
		**MT**	166 (49.1)		
		**TT**	89 (26.0)		
		**T**		0.51	0.43°
***eNOS***	**rs2070744**	**TT**	131 (38.3)		
		**TC**	147 (43.0)		
		**CC**	64 (18.7)		
		**C**		0.40	0.41°
	**rs1799983**	**GG**	166 (48.5)		
		**GT**	145 (42.4)		
		**TT**	31 (9.1)		
		**T**		0.30	0.36°
	**rs61722009**	**bb**	247 (72.2)		
		**ab**	85 (24.9)		
		**aa**	10 (2.9)		
		**a**		0.15	0.17°
***VEGFA***	**rs3025039**	**CC**	240 (70.2)		
		**CT**	88 (25.7)		
		**TT**	14 (4.1)		
		**T**		0.17	0.15*
	**rs1547651**	**AA**	255 (74.6)		
		**AT**	77 (22.5)		
		**TT**	10 (2.9)		
		**T**		0.14	0.13*
	**rs833058**	**CC**	109 (31.9)		
		**CT**	160 (46.8)		
		**TT**	73 (21.3)		
		**T**		0.45	0.39*
	**rs833061**	**TT**	99 (28.9)		
		**TC**	170 (49.7)		
		**CC**	73 (21.3)		
		**C**		0.46	0.38*
***HMOX1***	**rs3074372**	**LL**	150 (43.8)		
		**SL**	162 (47.4)		
		**SS**	30 (8.8)		
		**S**		0.31	-

There was a higher prevalence of TC+CC rs2070744 *eNOS* (74.6 vs 58.3%, p = 0.01) and GT+TT rs1799983 *eNOS* (64.8 vs 48.0%, p = 0.01) polymorphisms in infants with BPD compared to those without BPD (Table 4). Multivariate analysis indicates that these associations were statistically significant [TC+CC rs2070744: OR 1.89 (1.03–3.46), p = 0.04; GT+TT rs1799983: OR1.92 (1.09–3.39), p = 0.02] after adjustment for gestational age and birth weight (Table 5). The contemporary presence of TC or CC rs2070744 and GT or TT rs1799983 *eNOS* genotypes was associated with an increased OR for BPD (OR 2.26 (1.30–3.95), p = 0.004).

**Table 4:** Genotype distribution of the 12 polymorphisms in infants with or without RDS requiring MV and BPD.

			RDS			BPD		
Gene Symbol	db SNP	Genotypes	Non = 182 (%)	Yesn = 160 (%)	p	Non = 271 (%)	Yesn = 71 (%)	p
***ACE***	**rs1799752**	**II**	26 (14.3)	32 (20.0)		48 (17.7)	10 (14.1)	
		**ID+DD**	156 (85.7)	128 (80)	0.2	223 (82.3)	61 (85.9)	0.5
***ACE* Prom**	**rs4291**	**AA**	66 (36.3)	53 (33.1)		101 (37.3)	18 (25.4)	
		**AT+TT**	116 (63.7)	107 (66.9)	0.5	170 (62.7)	53 (74.6)	0.06
***AGTR1***	**rs5186**	**AA**	117 (64.3)	94 (58.8)		164 (60.5)	47 (66.2)	
		**AC+CC**	65 (35.7)	66 (41.3)	0.3	107 (39.5)	24 (33.8)	0.4
***AGT***	**rs699**	**MM**	48 (26.4)	37 (23.1)		73 (26.9)	12 (16.9)	
		**MT+TT**	134 (73.6)	123 (77.9)	0.5	198 (73.1)	59 (83.1)	0.08
***eNOS***	**rs2070744**	**TT**	71 (39.0)	60 (37.5)		113 (41.7)	18 (25.4)	
		**TC+CC**	111 (61.0)	100 (62.5)	0.8	158 (58.3)	53 (74.6)	**0.01**
	**rs1799983**	**GG**	90 (49.5)	76 (47.5)		141 (52.0)	25 (35.2)	
		**GT+TT**	92 (50.5)	84 (52.5)	0.7	130 (48.0)	46 (64.8)	**0.01**
	**rs61722009**	**bb**	133 (73.1)	114 (71.2)		197 (72.7)	50 (70.4)	
		**ab+aa**	49 (26.9)	46 (28.8)	0.7	74 (27.3)	21 (29.6)	0.7
***VEGFA***	**rs3025039**	**CC**	126(69.2)	114(71.2)		184(67.9)	56(78.9)	
		**CT+TT**	56(30.8)	46(28.8)	0.6	87(32.1)	15(21.1)	0.07
***VEGFA***	**rs1547651**	**AA**	143(78.6)	112(70.0)		202(74.5)	53(74.6)	
		**GG+GA**	39(21.4)	48(30.0)	0.06	69(25.5)	18(25.4)	0.9
***VEGFA***	**rs833058**	**CC**	52(28.6)	57(35.6)		89(32.8)	20(28.2)	
		**CT+TT**	130(71.4)	103(64.4)	0.1	182(77.2)	51(72.8)	0.4
***VEGFA***	**rs833061**	**TT**	54(29.7)	46(28.8)		76(28.0)	24(33.8)	
		**CT+CC**	128(70.3)	114(71.2)	0.8	195(72.0)	47(66.2)	0.3
***HMOX1***	**rs3074372**	**LL**	79 (43.4)	71 (44.4)		115 (42.4)	35 (49.3)	
		**SL+SS**	103 (56.6)	89 (55.6)	0.8	156 (57.6)	36 (50.7)	0.3

**Table 5:** Univariate and multivariate analysis for VEGF, ACT, *AGT*, *eNOS* polymorphisms.

*RDS*				
	**Univariate Analysis**		Multivariate Analysis*	
	**OR (95%CI)**	**p**	**OR (95%CI)**	**p**
**rs 1547651 *VEGFA* T**	1.57 (0.96–2.56)	0.07	1.47 (0.82–2.63)	0.2
***BPD***				
	**Univariate Analysis**		Multivariate Analysis*	
	**OR (95%CI)**	**p**	**OR (95%CI)**	**p**
**rs 4291 *ACE* Prom -240T**	1.75 (0.97–3.15)	0.06	1.61 (0.87–2.97)	0.13
**rs699 *AGT* 235T**	1.82 (0.93–3.58)	0.08	1.87 (0.92–3.84)	0.08
**rs2070744 *eNOS* -786C**	2.11 (1.17–3.79)	**0.01**	1.89 (1.03–3.46)	**0.04**
**rs1799983 *eNOS* 894T**	1.98 (1.15–3.41)	**0.01**	1.92 (1.09–3.39)	**0.02**
**rs3025039 VEGF 936T**	0.57 (0.30–1.06)	0.07	0.59 (0.31–1.12)	0.1
***ROP***				
	**Univariate Analysis**		Multivariate Analysis*	
	**OR (95%CI)**	**p**	**OR (95%CI)**	**p**
**rs1799983 *eNOS* 894T**	1.90 (1.00–3.71)	**0.05**	1.73 (0.83–3.64)	0.1
**rs833061 VEGF -460C**	0.66 (0.34–1.29)	0.2	0.74 (0.35–1.58)	0.4
**rs833058 *VEGFA* T**	1.63 (0.77–3.45)	0.2	1.28 (0.57–2.90)	0.5

We observed a trend toward a different prevalence of AT+TT rs4291 *ACE* polymorphism of the promoter region, MT+TT rs699 *AGT*, and CT+TT rs3025039 *VEGFA* polymorphisms in infants with BPD compared to those without BPD (Table 4), and of GT+TT rs1799983 *eNOS* in infants with ROP compared to those without ROP (Table 6). However, these associations disappeared after adjusting for gestational age and birth weight (Table 5).

**Table 6:** Genotype distribution of the 12 polymorphisms in infants with or without retinopathy of prematurity (ROP) and intraventricular hemorrhage (IVH).

			ROP			IVH		
Gene Symbol	db SNP	Genotypes	Non = 299 (%)	Yesn = 43 (%)	p	Non = 272 (%)	Yesn = 70 (%)	p
***ACE***	**rs1799752**	**II**	47 (15.7)	11 (25.6)		46 (16.9)	12 (17.1)	
		**ID+DD**	252 (84.3)	32 (74.4)	0.1	226 (83.1)	58 (82.9)	0.9
***ACE* Prom**	**rs4291**	**AA**	103 (34.4)	16 (37.2)		92 (33.8)	27 (38.6)	
		**AT+TT**	196 (65.6)	27 (62.8)	0.7	180 (66.2)	43 (61.4)	0.4
***AGTR1***	**rs5186**	**AA**	188 (62.9)	23 (53.5)		164 (60.3)	47 (67.1)	
		**AC+CC**	111 (37.1)	20 (46.5)	0.2	108 (39.7)	23 (32.1)	0.3
***AGT***	**rs699**	**MM**	74 (24.7)	11 (25.6)		70 (25.7)	15 (21.4)	
		**MT+TT**	225 (75.3)	32 (74.4)	0.9	202 (74.3)	55 (78.6)	0.4
***eNOS***	**rs2070744**	**TT**	116 (38.8)	15 (34.9)		105 (38.6)	26 (37.1)	
		**TC+CC**	183 (61.2)	28 (65.1)	0.6	167 (61.4)	44 (62.9)	0.8
	**rs1799983**	**GG**	151 (50.5)	15 (34.9)		135 (49.6)	31 (44.3)	
		**GT+TT**	148 (49.5)	28 (65.1)	**0.05**	137 (50.4)	39 (55.7)	0.4
	**rs61722009**	**bb**	214 (71.6)	33 (76.7)		197 (72.4)	50 (71.4)	
		**ab+aa**	85 (28.4)	10 (23.3)	0.5	75 (27.6)	20 (28.6)	0.9
***VEGFA***	**rs3025039**	**CC**	210(70.2)	30(69.8)		193(71.0)	47(67.1)	
		**CT+TT**	89(29.8)	13(30.2)		79(29.0)	33(32.9)	0.5
					0.9			
***VEGFA***	**rs1547651**	**AA**	221(73.9)	34(79.1)		203(74.6)	52(74.3)	
		**GG+GA**	78(26.1)	9(20.9)	0.5	69(25.4)	18(25.7)	0.9
***VEGFA***	**rs833058**	**CC**	99(33.1)	10(23.3)		87(32.0)	22(31.4)	
		**CT+TT**	200(66.9)	33(76.7)	0.1	185(68.0)	48(68.6)	0.9
***VEGFA***	**rs833061**	**TT**	84(28.1)	16(37.2)		76(27.9)	24(34.3)	
		**CT+CC**	215(72.9)	27(62.8)	0.2	196(72.1)	46(65.7)	0.3
***HMOX1***	**rs3074372**	**LL**	132 (44.1)	18 (41.9)		121 (44.5)	29 (41.4)	
		**SL+SS**	167 (55.9)	25 (58.1)	0.8	151 (55.5)	41 (58.6)	0.6

Gestational age and birth weight were not associated with any of the analyzed polymorphisms (Table 7).

**Table 7:** Gestational age and birth weight at according to genotype distribution of studied polymorphisms.

			Gestational Age at birth			Weight at birth		
Gene Symbol	db SNP	Genotypes	n	Median weeks (IQR Range)	p	n	Median grams (IQR range)	p
***ACE***	**rs1799752**	**II**	58	26.7 (25.3–28.3)		58	900 (730–1120)	
		**ID+DD**	284	27.6 (25.8–28.5)	0.2	284	872 (725–1077)	0.5
***ACE* Prom**	**rs4291**	**AA**	119	27.6 (25.8–28.9)		119	910 (730–1120)	
		**AT+TT**	223	27.4 (25.7–28.4)	0.5	223	865 (730–1070)	0.3
***AGTR1***	**rs5186**	**AA**	211	27.4 (25.4–28.7)		211	870 (707.5–1082.5)	
		**AC+CC**	131	27.6 (26.0–28.4)	0.9	131	897.5 (747.5–1100)	0.4
***AGT***	**rs699**	**MM**	85	27.2 (25.7–28.7)		85	910 (760–1125)	
		**MT+TT**	257	27.6 (25.5–28.4)	0.6	257	871 (711.2–1080)	0.3
***eNOS***	**rs2070744**	**TT**	131	27.4 (26.3–28.5)		131	940 (754–1110)	
		**TC+CC**	211	27.6 (25.4–28.6)	0.6	211	865 (720–1075)	0.2
	**rs1799983**	**GG**	166	27.7 (25.9–28.7)		166	900 (735–1120)	
		**GT+TT**	176	27.4 (25.4–28.4)	0.2	176	871 (720–1060)	0.2
	**rs61722009**	**Bb**	247	27.6 (25.9–28.6)		247	890 (750–1100)	
		**ab+aa**	95	27.1 (25.3–28.3)	0.3	95	870 (700–1053.7)	0.3
***VEGFA***	**rs3025039**	**CC**	240	27.6 (25.6–28.6)		240	867.5 (720–1097.5)	
		**CT+TT**	102	27.3 (25.8–28.4)	0.9	102	910 (760–1082.5)	0.4
***VEGFA***	**rs1547651**	**AA**	255	27.6 (25.7–28.7)		255	880 (730–1100)	
		**AT+TT**	87	27.2 (25.6–28.3)	0.1	87	860 (720–1050)	0.4
***VEGFA***	**rs833058**	**CC**	109	27.7 (26.1–28.6)		109	890 (755–1100)	
		**CT+TT**	233	27.4 (25.4–28.6)	0.5	233	880 (720–1080)	0.3
***VEGFA***	**rs833061**	**TT**	100	27.2 (25.4–28.3)		100	872.5 (742.5–1047.5)	
		**CT+CC**	242	27.6 (26.0–28.6)	0.2	242	890 (720–1110)	0.6
***HMOX1***	**rs3074372**	**LL**	150	27.6 (25.7–28.6)		150	915 (745–1105)	
		**SL+SS**	192	27.6 (25.8–28.6)	0.9	192	865 (720–1085)	0.3

According to the multivariate analysis, adjusted for gestational age and birth weight, we found that:

1) *VEGFA* haplotypes increase (ACCC, ACTC, ACTT, ATCC, TCCT) or decrease (ACCT, TCCC) birth weight (Table 8); 2) the *eNOS* haplotype (TaG) decreases gestational age (Table 8). We also found that the: 1) *VEGFA* haplotype (ACTT) decreases the risk of RDS and increases the risk of BPD (Table 9); 2) the *VEGFA* haplotype (TCCT) decreases the risk of ROP and increases the risk of IVH (Tble 9); 3) the rare (CaT and TaT) and CbT eNOS haplotypes were significantly associated with different effects (risk for RDS and BPD and protection from IVH and ROP) on clinical prematurity complications (Table 9).

**Table 8:** Haplotypes of VEGF and *eNOS* genes significantly associated with weight and gestational age at birth.

Gene Symbol	Haplotype	Frequency	Coefficient	Standard Error	p-value
		**Weight at birth**			
***VEGFA***					
	ACCC	0.232	37.2	5.58	**<0.0001**
	ACCT	0.065	-15.2	1.23	**<0.0001**
	ACTC	0.103	22.2	2.08	**<0.0001**
	ACTT	0.012	35.2	0.19	**<0.0001**
	ATCC	0.022	40.6	0.39	**<0.0001**
	TCCC	0.101	-47.9	1.91	**<0.0001**
	TCCT	0.029	73.2	0.46	**<0.0001**
SNP1 = rs1547651, SNP2 = rs833058, SNP3 = rs833061, SNP4 = rs3025039					
		**Gestational age at birth**			
***VEGFA***					
	ACCC	0.232	0.33	0.19	0.08
***eNOS***					
	TaG	0.021	-1.33	0.53	**0.01**
SNP1 = rs2070744, SNP2 = **rs61722009**, SNP3 = rs1799983					

**Table 9:** Haplotypes of the VEGF and *eNOS* genes significantly associated with the adverse outcomes.

Gene Symbol	Outcome	Haplotype	Frequency in subjects without adverse outcome	Frequency in subjects with adverse outcome	Coefficient	Standard Error	p
**VEGF**							
	**BPD**	ACTT	0.0009	0.038	0.8	0.01	**<0.0001**
	**RDS**	ACTT	0.013	0.008	-0.08	0.01	**<0.0001**
	**ROP**	TCCT	0.036	NA	-0.1	0.07	**0.03**
	**IVH**	TCCT	0.019	0.064	0.3	0.1	**0.02**
SNP1 = rs1547651, SNP2 = rs833058, SNP3 = rs833061, SNP4 = rs3025039							
***eNOS***							
	**BPD**	CbT	0.197	0.294	0.09	0.04	**0.02**
SNP1 = rs2070744, SNP2 = **rs61722009**, SNP3 = rs1799983							

**NA**: not applicable.

## Discussion

In this study we evaluated the possible association between *VEGFA*, *eNOS*, *AGT*, *AGTR1*, *ACE*, and *HMOX-1* gene polymorphisms and the development of the most frequent prematurity complications, namely RDS requiring MV, BPD, IVH and ROP in a large population of preterm infants. Our data demonstrate for the first time that the T allele of the rs1799983 *eNOS* polymorphism and confirm that C allele of the rs2070744 *eNOS* polymorphism are independent predictors of an increased risk of BPD in preterm infants. We also discovered that haplotypes of *VEGFA* and eNOS genes may independently affect birth weight and gestational age.

Although further associations emerged from the univariate analysis, these correlations disappeared after data adjustment for gestational age and birth weight, with the exception of TC+CC rs2070744 *eNOS* and GT+TT rs1799983 *eNOS* genotypes which were independent risk factors for BPD. We speculated that the lack of correlations of the different polymorphisms “per se” was due to the multifactorial pathogenesis of studied prematurity complications which were mainly governed by gestational age [26–30].

The role of the rs2070744 *eNOS* polymorphism in the pathogenesis of RDS and BPD has been previously investigated in a cohort (n = 124) of preterm infants by Vannemreddy et al. [6]. They found that the status of rs2070744 allele C carrier(TC+CC) was significantly more frequent in infants with RDS, BPD, and IVH compared to control patients (15.3 vs. 7.3%, p = 0.03) and represents a significant risk factor for the development of these pathologies [6] Our data are in partial agreement with these findings because we did not observe any relationship between the polymorphism and the risk of severe RDS and IVH, but we could confirm that TC+CC rs2070744 *eNOS* genotypes are a risk factor for BPD. Previous reports have demonstrated that TC heterozygotes and CC homozygotes for the rs2070744 eNOS polymorphism increase the risk of cardiovascular diseases in adults and decreases NO levels [6,31]. Considering the crucial role of NO in promoting lung angiogenesis during the fetal/neonatal period [2,32,33], we can reasonably speculate that the C allele of the polymorphism increases the risk of BPD through the decrease of endothelial NO synthesis that impairs lung vessel growth and in turn alveologenesis.

Similarly, the role of the rs1799983 *eNOS* polymorphism has been demonstrated in the pathogenesis of cardiovascular diseases in adults [34,35], but has never been investigated in the pathogenesis of prematurity complications. We found that GT+TT rs1799983 *eNOS* genotypes increase the risk of BPD. It has been demonstrated that the T allele carrier status is associated with reduced function of the enzyme and decreased NO production by endothelial cells compared to GG homozygous subjects [36,37]. Therefore, these polymorphisms might also favor BPD development through a negative effect on lung vessels and alveolar growth.

These considerations seem to be confirmed by the results of Ballard’s large randomized controlled study demonstrating that prolonged inhaled NO treatment increases survival without BPD in high- risk preterm infants, although its routine use can not be recommended [38].

Interestingly, the multivariate analysis (adjusted for gestational age and birth weight) of the polymorphism combinations by haplotype analysis of the polymorphisms localized in the same gene showed that haplotypes of *VEGFA* increase or decrease birth weight and one haplotype of *eNOS* decreases gestational age. Moreover, the *VEGFA* haplotype increases the risk of IVH and BPD or decreases the risk of RDS and ROP, while the *eNOS* haplotype increases the risk of RDS and BPD or decreases the risk of IVH and ROP. Although it is difficult to explain these different effects, if we consider the crucial role of *VEGFA* [1,2] and NO [5,6] on fetal and neonatal vessel growth and protection, we may speculate that protective haplotypes might enhance their beneficial effects, and promote adequate tissue vascularization and perfusion, while unfavorable combinations might attenuate them and induce inadequate vessel development and blood flow.

Haplotype analysis allow us to identify alleles in which polymorphisms *per se* associated with the diseases are included, and helps to better identify subjects who are either at risk or not (e.g. *VEGFA* haplotypes for RDS, BPD, ROP, and IVH). Thus, our findings suggest that different SNPs in *VEGFA* and *eNOS* genes of low frequency might change the patients’ susceptibility to prematurity complications by altering either their function or their expression.

The limitations of our study include the relatively small size of the study which could affect the demonstration of correlation between studied complications and polymorphisms; the lack of measurement of infants’ *VEGFA* levels and NOS activity which precludes the possibility of relating specific polymorphisms and haplotype combinations with their expression in our patients. Thus, our findings do not allow us to demonstrate definitively whether the aforementioned SNPs and haplotypes are themselves responsible for their beneficial or detrimental effects in our population or whether they reflect linkage disequilibrium with variation in the remainder of the genes or perhaps in a nearby flanking gene. However, given the homogeneity of our population, it is unlikely that the observed associations are a statistical artefact resulting from a population substructure.

## Conclusions

We confirm that the C allele of rs2070744 *eNOS* polymorphism is an independent predictor of an increased risk of BPD in preterm infants and demonstrate for the first time the same thing for the T allele of the rs1799983 *eNOS* polymorphism. We also find that haplotypes of *VEGFA* and eNO genes may independently affect birth weight and gestational age, and act as protecting or risk markers for prematurity complications. These results could be useful in identifying patients at high risk of developing prematurity complications in whom prevention strategies would be particularly indicated.

## Supporting Information

S1 Appendix Database of clinical characteristics and polymorphisms of studied infants. (XLS)
